# Utilization of growth monitoring and promotion services and undernutrition of children less than two years of age in Northern Ghana

**DOI:** 10.1186/s40795-023-00729-6

**Published:** 2023-06-22

**Authors:** Benjamin Baguune, Dramani Mahama Aminu, Emmanuel Bekyieriya, Martin Nyaaba Adokiya

**Affiliations:** 1 School of Hygiene, Ministry of Health, Box TL 88, Tamale, Ghana; 2 Department of Nutritional Sciences, School of Allied Health Sciences, University for Development Studies, Tamale, Ghana; 3 Department of Epidemiology, Biostatistics and Disease Control, School of Public Health, University for Development Studies, Tamale, Ghana

**Keywords:** Maternal, Child, GMP, Feeding practice, Undernutrition, Ghana

## Abstract

**Background:**

Child undernutrition is a major public health problem and an important indicator of child’s health. Adequate nutrition is critical for a child’s growth and development. Growth monitoring and promotion (GMP) services is a nutrition intervention aimed at improving the nutritional status of children. We assessed the utilization of growth monitoring and promotion services and nutritional status of children less than two years of age in northern Ghana.

**Methods:**

This was a descriptive cross-sectional study that involved face-to-face interviews among 266 mothers with children < 2 years of age attending child welfare clinics (CWC). We also collected anthropometric measurements. Descriptive statistic was performed and data presented as percentage. The nutritional status of children was classified as underweight (weight-for-age z score < -2 standard deviations), stunted (length-for-age Z score < − 2) and wasted (weight-for-length z score < -2) while utilization of GMP services was based on attendance to CWC and ability to interpret different growth curves. Chi square test was used to determine the relationship between utilization of GMP services and nutritional status of children at an alpha of 0.05.

**Results:**

The prevalence of undernutrition shows that, 18.6% of the children were underweight, 14.7% were stunted and 7.9% were wasted. About 60% of the mothers accessed GMP services regularly. Less than half of the mothers were able to interpret the children’s growth curve correctly: falling growth curve (36.8%), flattening growth curve (35.7%) and rising growth curve (27.4%). In combining children < 6 and 6–23 months of age, only one-third (33.1%) of mothers practiced appropriate infant and young child feeding. Regular GMP services was found to have a statistically significant relationship with underweight (P < 0.001), stunting (P = 0.006) and wasting (P = 0.042).

**Conclusion:**

The level of undernutrition remains high and child feeding practices is poor. Maternal utilization of GMP services is also low in the study area. Similarly, ability to interpret the child’s growth curve appropriately persist as a challenge among women. Thus, attention is needed to improve utilization of GMP services to address child undernutrition challenges.

**Supplementary Information:**

The online version contains supplementary material available at 10.1186/s40795-023-00729-6.

## Introduction

Undernutrition remains the world’s most serious health problem and a major contributor to child mortality [1]. Child undernutrition is responsible for > 50.0% of all childhood deaths [2]. Globally, it is estimated that, 25.6% of children < 5 years of age are stunted with prevalence rates > 40% in many developing countries [3]. Also, it is estimated that, 43.0% of children < 5 years in developing countries are prone to falling short of their life potentials due to poor nutritional situations they faced in their early formative years [4]. The inadequacy of growth is high among children < 24 months of age [5]. The period from birth until two years of age is considered a critical stage for maximum nutrition care to enhance optimum growth. This is the basis for global initiatives toward child care [6]. Child undernutrition is an important public health problem in Ghana. According to 2014 Ghana Demographic and Health Survey (GDHS) report, 19.0% of children < 5 years of age were stunted, 11.0% were underweight and 5.0% were wasted. In northern Ghana, 33.1% of children were stunted, 20.1% were underweight and 6.3% were wasted. Similarly, stunting was between 8.0% and 21.9%, underweight was between 4.2% and 14.6% and wasting was between 6.9% and 10.6% among children less than 2 years of age in Ghana [7].

Growth monitoring (GM) is not an intervention in itself but a process that best defines the children’s health and nutritional status. It provides a measurement on the quality of life of the entire population [8]. On the other hand, the promotion aspect of GMP covers a range of activities such as those focusing on health education, counseling, referral and other actions based on the outcome of the measurement. The growth pattern of a child is determined by comparing his/her growth indices with that of a reference child of the same age and sex [8]. In Ghana, GMP services are implemented at both community and health facility levels through the primary health care system to improve child nutritional status. GMP is a prevention activity comprised child GM linked with promotion that increases awareness about child growth; improves caring practices and increases demand for other services. It also serves as the core activity in an integrated child health and nutrition program [9]. Globally, growth chart is used as a tool to monitor child’s growth and determine the nutritional status of children. GM uses weight-for-age, weight-for-height and height-for-age as indicators to determine the nutritional status of children. The growth monitoring outcomes are complemented with targeted counseling for children who have some growth deficits [10]. The GM also provides opportunity for communication between health workers and caregivers concerning the wellbeing of their children [11]. Regular monitoring of growth helps in early detection of danger warning signs and conditions that affect growth [12]. In Ghana, children are routinely weighed on a monthly basis. Rates of utilization or attendance of GMP sessions vary in Sub-Saharan Africa (SSA) and in other developing countries [13]. In Senegal, 70.0% of children attended monthly GMP provided by a non-governmental organization, whereas 35.0% patronized the government facility [13]. Another study conducted in Ethiopia among children aged 0–24 months of age revealed a GMP utilization rate of 16.9% [14]. In the Ga West District of Ghana, Agbozo et al. [11] reported 13.6% of caregiver-child pairs obtaining more than 9 visits in the last 12 months. Gyampo et al. reported 70.0% of mothers not missing scheduled child welfare clinic session in a study conducted in the Accra Metropolitan Area of Ghana [15].

Despite several efforts to reduce child undernutrition through GMP services in Ghana, prevalence of undernutrition remains high among children < 5 years of age, particularly in northern Ghana [7]. In addition, GMP has been implemented in Ghana since the 1970s, evidence regarding the utilization of GMP services among mothers with their children is limited. In a review of the literature, we found some studies [11, 15–17] on GMP conducted in Ghana but these studies did not provide information on utilization of GMP services and child nutritional status. The current study assessed the utilization of growth monitoring and promotion services and nutritional status of children less than two years of age in Tamale Metropolis of northern Ghana.

## Materials and methods

### Study design and setting

We employed a cross-sectional descriptive design to collect data from mothers with children less than two years of age. The study was conducted in the Tamale Metropolis of Northern Region, Ghana. There are six (6) Metropolis in Ghana and the Tamale Metropolis is the only one in the north of the country. Currently, the north of Ghana is composed of five (5) regions namely: Upper East, Upper West, Northern, North-East and Savannah Regions. The Metropolis is located in the central part of the Northern Region with Tamale being the capital. It has an estimated land size of 646.90180 square kilometre and a population of 233,252 [18]. According to the database of the District Health Information Management System, 64,641 women are in their reproductive age and 10,769 children are less than two years of age.

### Study participants and sampling

The Metropolis is demarcated into four sub-metropolises for the administration of health services [18]. The target population were women of childbearing age (15–49 years) with children < 2 years of age. The main criteria for inclusion were: (1) a mother who has a child less than two years of age; (2) a mother who was present at the time of the survey; (3) child of singleton birth, (4) no obvious signs of illness and (5) a mother who consented to participate in the study. The children chosen were within the critical stage of the first 1,000 days of a child’s life. We used the formula (N = Z^2^pq/e^2^) by Cochran (1963) to calculate the sample size for mother-child pairs for the study. We used the highest prevalence of underweight (20%) among children less than two years in Northern Ghana [7]. In using a 95% confidence interval (CI) and 5% margin of error, a sample size of 246 was obtained. A 10% nonresponse rate was computed and a final sample size of 270 was determined.

The study was a community-based study and participants were selected using child welfare clinic (CWC) registers. A list of all health facilities providing GMP services in the sub-metropolis was compiled. A random sampling method used to select one health facility from each sub-metropolis for the study. The four health facilities selected comprised of one reproductive and child health unit, one health center and two clinics. A sampling frame containing a list of 8186 (Table 1) mother-child pairs less than 2 years old along with their date of birth and house number was obtained from the CWC registers from the selected health facilities. The households list from each health facility (sub-metro) were coded by numeric code and a total of 270 households were picked by lottery method based on proportional allocation (Table 1) to the number of CWC attendance from each health facility (sub-metro). All households were visited for the data collection. We used the house numbers obtained from the CWC register to locate the households. If we found two children who were less than 2 years old within the household, a child with a smaller age was taken. If we found twins within the households, we picked either of the children using the lottery method. If an eligible mother was absent during the data collection period, a revisit was done three times, and mothers absent on the third visit were considered non-respondent. Overall, 266 interviews were conducted comprising of 122 mothers with children aged < 6 months and 144 women with children aged between 6 and 23 months of age.

**Table 1:** Mothers attending child welfare clinics in Tamale Metropolis

Sub-metropolis	Health facility	CWC attendance	Weighted sample size
Tamale central	RCH Unit	2721	91
Builpela	Builpela Health Center	2049	68
Vittin	Kotinli Clinic	1984	64
Nyohini	Nyohini Clinic	1432	45
**Total**		**8186**	**270**

Legend

CWC: Child Welfare Clinic

RCH: Reproductive and Child Health

### Data collection tool and procedure

A structured questionnaire was used for the data collection (Supplementary file 1). It was developed based on the WHO-UNICEF guidelines on infant feeding [19, 20]. The components of the questionnaire were mother and child demographic characteristics, utilization of GMP services, Child GM information and feeding practices. The tools were pretested in a health facility providing GMP services but was not selected to be part of the main study.

Data was collected through face-to-face interviews with mothers and measurements of weight and height of children. The demographic characteristics and GMP information were gathered through the face-to-face interviews. A 24-hour recall approach was also used to assess exclusive breastfeeding, minimum dietary diversity, minimum meal frequency, continued breastfeeding, and bottle feeding. Child’s lifetime historical recall approach was used to elicit information on early initiation of breastfeeding and timely initiation of complementary feeding.

Child anthropometric measurements were taken according to WHO guidelines [19]. We used Beurer digital scale to measure the weight of the children and recorded it to the nearest 0.1 kg. We also used a locally manufactured infantometer with a fixed headboard and a movable footboard to measure the recumbent length of children. The length was recorded to the last completed 1 cm. Weight and length measurements were duplicated and the average recorded. Two final year students pursuing Bachelor of Science in Nursing at the University for Development Studies, Tamale, conducted the interviews privately. The interviews were conducted in Dagbani and English languages. The data collectors received training on the study’s purpose and data collection methodology. The study was conducted from January - February, 2018.

### Operational definitions

#### Growth monitoring

This is regular monthly weighing of a child and comparing the results with a standard to detect abnormal growth rate combined with some actions when detected [21].

#### Regular GMP attendance

This was measured when a child has a history of GMP services utilization in respect to age such as, at least once for 0 months, two times for 1–3 months, five times for 4–11 months, and four times per year for 12–23 months and the finding should be plotted /recorded on the child health record card [10].

#### Irregular attendance

Any child who did not meet the criteria for regular attendance outlined above.

#### Nutritional status

Outcome of an assessment of the physiological status of the body based on anthropometric measurement of its height and weight.

### Data processing and analysis

Data was checked for completeness and accuracy daily. We performed descriptive analysis using the Statistical Package for Social Science software for Windows, version 20. Utilization of GMP services was dichotomized (regular attendance and irregular attendance) and analyzed as frequencies and percentages.

Infant and young child feeding (IYCF) indicators were defined and estimated per the updated WHO-UNICEF– recommended guidelines published in 2021 [20]. The IYCF indicators analyzed in this study were as follows: early initiation of breastfeeding (proportion of children born in the last 24 months who are breastfed within 1 h of birth), exclusive breastfeeding up to 6 months (proportion of infants 0–5 months of age who are fed exclusively with breast milk), introduction of solid, semi-solid, or soft foods (proportion of infants 6–8 months of age who receive solid, semi-solid, or soft foods), minimum dietary diversity (MDD) (proportion of children 6–23 months of age who receive foods from ≥ 5 food groups. minimum meal frequency (MMF) (proportion of children 6–23 months of age who receive solid, semi-solid, or soft foods the minimum number of times or more) [20].

Child anthropometric data are expressed by age and sex appropriate z-scores for weight-for-age (WAZ), weight-for-height/length (WHZ) and height-for-age (HAZ). The children were grouped into normal z-scores for all indicators ≥-2 standard deviation, underweight (WAZ <-2 score), wasting (WHZ <-2 score) and stunting (HAZ <-2 score). Using frequency and percentage analysis, the child nutritional status was classified into normal as against underweight, stunted or wasted [22]. Chi square test was used to identify the relationship between utilization of GMP services and nutritional status of children at an alpha of 0.05.

## Results

### Demographic characteristics

In total, 266 mothers with children less than 2 years were enrolled. About 85% of the participants were between 18 and 34 years of age. More than half (57.5%) of the participants had either no education (45.9%) or only primary education (11.6%). Nearly all (94.7%) of the mothers were married. About half (42.8%) of the mothers were traders and more than half (56.0%) had 1–2 children. Less than half (46.0%) of the children were < 6 months of age. More than half (53.8%) of the children were males (Table 2).

**Table 2:** Socio-demographic characteristics of mothers with children < 2 years (n = 266)

Characteristics of respondents	Frequency	Percentage (%)
**Age of mother (years)**		
<18	2	0.8
18–34	226	85.0
≥ 35	38	14.2
**Mothers’ educational level**		
No education	122	45.9
Primary	31	11.6
Secondary	81	30.5
Tertiary	32	12.0
**Marital status of mothers**		
Single	14	5.3
Married	252	94.7
**Mothers’ occupation**		
Unemployed	103	38.7
Trader	144	42.8
Artisan	14	5.3
Salary workers	35	13.2
**Ethnicity of mothers**		
Dagomba	224	84.2
Gonja/Mamprusi/Akan	11	4.2
Moshi/Bimoba	29	11.6
**Parity**		
1–2	149	56.0
3–4	83	31.2
5+	34	12.8
**Age of child (months)**		
< 6	122	46.0
6–11	94	35.3
12–23	50	18.7
**Sex of children**		
Male	143	53.8
Female	123	46.2

### Utilization of GMP Services

About three-fifths (59.8%) of the mothers were accessing regular monthly GMP services at the CWC (Table 3). Traveling of mothers (68.0%) was the main reason for irregular GMP attendance. The analysis showed that about one-third of mothers were able to interpret falling (36.8%) and flattening (35.7%) growth curves while a little above one-quarter (27.4%) interpreted rising growth curve correctly. The main action taken by mothers was taking the child to a hospital (33.8% for falling growth curve and 29.3% for flattening growth curve).

**Table 3:** Utilization of GMP Services (n = 266)

Variable	Frequency	Percentage (%)
**Monthly GMP attendance**		
Regular attendance	159	59.8
Irregular attendance	107	40.2
**Barriers to GMP attendance**		
Travelled	181	68.0
Work	51	19.2
Others	34	12.8
**Ability to interpret falling growth curve**		
Yes	98	36.8
No	168	63.2
**Ability to interpret flattening growth curve**		
Yes	95	35.7
No	171	64.3
**Ability to interpret rising growth curve**		
Yes	73	27.4
No	193	72.6
**Response during growth curve falling**		
Improve feeding	57	21.4
Take child to hospital	90	33.8
Seek nutritional care	7	2.6
Don’t know	112	42.2
**Response during growth curve flattening**		
Improve feeding	11	4.1
Take child to hospital	78	29.3
Give more breastmilk	54	20.3
Don’t know	123	46.2
**Response during growth curve rising**		
Continued breastfeeding and improved care	154	57.9
Don’t know	112	42.1

Legend

GMP: Growth Monitoring and Promotion

### Nutritional status of children less than two years of age attending CWC services

The nutritional status of children was classified as: underweight, stunting and wasting as summarized based on the WHO classification. Based on the anthropometric measurements, 18.6% of the children were underweight, 14.7% were stunted and 7.9% were wasted.

### Prevalence of IYCF core indicators

Table 4 illustrates the prevalence of WHO-UNICEF (2021) – recommended IYCF core indicators [20]. From the 266 children aged 0–23 months, 83.8% were breastfed within 1 h after birth and the prevalence of exclusive breastfeeding was 72.1% for infants aged 0–5 months. Nearly 4 out of 5 (84.6%) children were introduced to solid, semi-solid, or soft foods at 6–8 months of age. While majority (86.1%) of children received minimum meal frequency, only 11.8% of them received minimum dietary diversity.

**Table 4:** Prevalence of the WHO-UNICEF–recommended (2021) infant and young child feeding indicators

Variable	Frequency	Percentage (%)
**Early initiation of breastfeeding** ^1^		
Yes	223	83.8
No	43	16.2
**Exclusive breastfeeding up to 6 months** ^**2**^		
Yes	88	72.1
No	34	27.9
**Introduction of solid, semi-solid, or soft food** ^**3**^		
Yes	22	84.6
No	4	15.4
**Minimum meal frequency** ^**4**^		
Yes	124	86.1
No	20	13.9
**Minimum dietary diversity** ^**5**^		
Yes	17	11.8
No	127	88.2

^**1**^ percentage of children born in the last 24 months who were put to the breast within one hour of birth

^**2**^ percentage of infants 0–5 months who were fed exclusively with breast milk during the previous day

^**3**^ percentage of infants 6–8 months who consumed solid, semisolid or soft foods during the previous day

^**4**^ percentage of children 6–23 months who consumed solid, semi-solid or soft foods (but also including milk feeds for non-breastfed children) at least the minimum number of times during the previous day

^**5**^percentage of children 6–23 months who consumed foods and beverages from at least five out of eight defined food groups during the previous day

Chi square test was used to determine the relationship between utilization of GMP services and nutritional status of children at a statistically significant alpha-level of 0.05 (Table 5). Overall, GMP service utilization was related with the three nutritional status indicators (underweight, stunting and wasting). GMP service utilization was found to have a statistically significant relationship with underweight (P < 0.001), stunting (P = 0.006) and wasting (P = 0.042).

**Table 5:** Relationship between utilization of GMP services and nutritional status of children less than 2 years (n = 266)

Indicator	GMP attendance		Total n (%)	Test statistic
	Irregular n (%)	Regular n (%)		
**Weight-age-Z scores**				
Normal	71 (32.9)	145 (67.1)	217 (100.0)	*χ*^*2*^ = 26.058, p < **0.001**
Underweight	36 (72.9)	13 (27.1)	49 (100.0)	
**Height-age-Z scores**				
Normal	83 (36.7)	143 (63.3)	226 (100.0)	*χ*^*2*^ = 7.590, p = **0.006**
Stunted	24 (60.5)	16 (39.5)	40 (100.0)	
**Weight-height-Z scores**				
Normal	93 (38.0)	152 (62.0)	245 (100.0)	*χ*^*2*^ = 4.091, p = **0.042**
Wasted	12 (57.1)	9 (42.9)	21 (100.0)	

## Discussion

We sought to assess the utilization of growth monitoring and promotion services and nutritional status of children < 2 years of age in northern Ghana. This study also assessed IYCF practice among mothers of children less than 2 years of age in the Tamale metropolis using the WHO-UNICEF indicators. Nearly all mothers were older than 18 years of age. About six out of every ten mothers had either no formal education or only primary education. The findings of the current study confirm similar conclusions that northern Ghana has low levels of literate population compared to the southern part of the country [7]. We found that the majority of the mothers were married. The high level of married mothers in this study is likely due to geographical area and the cultural/religious values. About half of the mothers were traders/artisans. In Muslim dominated communities, petty trade is a major activity for women [7]. Often, the extreme poor in such populations are engaged in artisan activities for income generation. The majority of the mothers had one or two children. This is very important for child growth and development. This is in contrast to the findings in the 2014 GDHS where more than half of the women in northern region had more than six children [7].

The effective utilization of GMP services is critical to child nutritional status. The focus of GMP services is to affect family-level decision making on child feeding. For health workers, it provides an opportunity to assess child health status and offer counseling on healthy feeding, whereas for mothers, they acquire knowledge about the growth of their children and how to improve on it [9, 23]. The current study found that underweight and stunting were lower while wasting was higher in comparison to the 2014 Ghana Demographic and Health Survey findings which shows that, 20.1% of children under five years were underweight, 33.1% stunted and 6.3% wasted in the northern region [7]. The nutritional status of children in the study area is generally poor. There have been similar findings about poor nutritional status in developing countries [24]. Black et al., (2008) found in their study that, developing countries bear the highest burden of under-nutrition in spite of the advancement made in nutrition worldwide [25]. Reasons for these findings are not clear but we think that, inadequate infant feeding practices coupled with infections are contributory factors. Unlike the Southern sector in Ghana, northern region is one of the poorest regions in the country with a single but short rainy season. This generally affects household food security and dietary diversity particularly in the dry season. It also affects farming, household income and food supply. These could contribute to the nutritional situation found in the current study.

A slight majority of the mothers were classified as regular attendants. This was similar to studies conducted in Kwazulu Natal and Uganda, which found that, 67% and 59% of mothers were regular attendants respectively [26]. However, this finding is higher than a previous study conducted by Feleke et al. 2017 who reported that the utilization rate of GMP services was 16.9% in Ethiopia [14]. The current finding may suggest a well-implemented intervention in Ghana compared to Ethiopia.

In order to reduce malnutrition in a developing country like Ghana, safe, adequate and acceptable infant and child feeding practice is vital. For this reason, WHO and UNICEF have recommended core infant feeding practices. In the current study, early initiation of breastfeeding, exclusive breastfeeding, minimum feeding frequency and introduction of solid, semisolid, or soft foods between 6 and 8 months of age were encouraging. These practices are likely to contribute to the reduction in undernutrition. The current study’s prevalence on appropriate IYCF practice is higher than a study conducted in Ethiopia’s Shashamane Woreda, where a few of the caregivers practiced appropriate IYCF [27]. These good practices could be due to the considerably increase in number of mothers seeking maternal health care as a result of the country’s ongoing promotion of free maternal services, providing a favorable chance for health professionals to promote IYCF practice. Even though the WHO guideline on IYCF practice [28] does not specify minimum standards to be met or the percentage that should be considered for public health importance, it is understandable that all children less than 2 years should follow all recommended feeding practices. However, this current study found that, about 12% of children 6–12 month in the study metropolis maintained Minimum Dietary Diversity (MDD). Dietary diversity is a proxy for adequate micronutrient density of foods. Food intake from at least five of the recommended food groups was assessed in children aged 6–23 months as minimum dietary diversity. This study’s minimum food diversity (11.8%) was lower than the national prevalence [7], as well as, findings of other studies conducted in Bangladesh (41.5%) and Sri Lanka (71.1%) [29, 30]. The reasons for this disparity could be attributed to the single and short annual rainy season in the northern region coupled with the general poor living conditions as compared to the southern part of the country. This can affect household food security and dietary diversity particularly in the dry season. This situation could be a contributory factor to the nutritional situation found in the current study and thus, urgent nutrition actions are needed to improve upon the current findings in the study metropolis.

We found that, regular utilization of GMP services is related with child undernutrition. In a related study conducted in the Volta region by Appoh and Kerkling also found that GMP and infant feeding practices have significant association with nutritional status of children [17]. In addition, in a prognostic model analysis, it was found that, an increase by 10.0% in GM participation was attributed to a 4.7% decrease in the prevalence of poor weight gain among infants aged 0–11 months, and a 2.9% decrease in prevalence of poor weight gain in those aged 12–23 months [19]. This relationship between GMP service utilization and nutritional status of children may be due to the targeted nutritional counseling and health education activities at the CWC sessions, but authors recommend further studies that could provide more explanation.

### Limitations of study

The study has some limitations. Data for this study were collected one time and thus, a probable change in child nutritional status in relation to attendance over time may have been missed. Additionally, the level of analysis was not detailed enough to make any statistical inferences. However, we believe that the findings reflect the prevailing situation among the participants in the study site.

## Conclusion

We found that, the level of undernutrition remains high and child feeding practices is poor and maternal utilization of GMP services was low in the study area. Similarly, ability to interpret the child’s growth curve appropriately persist as a challenge among women. The high child undernutrition and other feeding challenges maybe attributed to the low patronage of GMP services in the study area. Thus, attention is needed to improve knowledge and utilization of GMP services and to address breastfeeding and complementary feeding challenges.

## Electronic supplementary material

Below is the link to the electronic supplementary material.

Supplementary Material 1

## Data Availability

The dataset used and/or analyzed during the current study are available from the corresponding author on reasonable request.

## References

[1] World Bank. Repositioning Nutrition as Central to Development; A Strategy for Large Scale Action. Washington DC, USA; 2006.

[2] Asfaw M, Wondaferash M, Taha M, Dube L (2015). Prevalence of undernutrition and associated factors among children aged between six to fifty nine months in Bule Hora district, South Ethiopia. BMC Public Health.

[3] Black RE, Victora CG, Walker SP, Bhutta ZA, Christian P, de Onis M (2013). Maternal and child Nutrition Study Group. (maternal and child undernutrition and overweight in low-income and middle-income countries. Lancet (London England).

[4] Daelmans B, Darmstadt GL, Lombardi J, Black MM, Britto PR, Lye S (2017). Lancet Early Childhood Development Series Steering Committee. Early childhood development: the foundation of sustainable development. Lancet (London England).

[5] Lundeen EA, Stein AD, Adair LS, Behrman JR, Bhargava SK, Dearden KA (2014). COHORTS investigators: height-for-age z scores increase despite increasing height deficits among children in 5 developing countries. Am J Clin Nutr.

[6] Prentice AM, Ward KA, Goldberg GR, Jarjou LM, Moore SE, Fulford AJ (2013). Critical windows for nutritional interventions against stunting. Am J Clin Nutr.

[7] Ghana Demographic and Health Survey, Key indicators A. 2014. Retrieved October 18, 2017, from https://scholar.google.com/scholar?q=Ghana+Demographic+and+Health+ Survey.+Key + indicators,+Accra.+2014&hl = en&as_sdt = 0&as_vis = 1&oi = scholart.

[8] De Onis M, Branca F (2016). Childhood stunting: a global perspective. Matern Child Nutr.

[9] Griffiths M, Rosso JD (2007). Growth Monitoring and Promotion of healthy young child growth: evidence of effectiveness and potential to prevent malnutrition.

[10] Ashworth A, Shrimpton R, Jamil K (2008). Growth monitoring and promotion: review of evidence of impact. Matern Child Nutr.

[11] Otoo GE, Lartey AA, Pérez-Escamilla R (2009). Perceived incentives and barriers to exclusive breastfeeding among periurban ghanaian women. J Hum Lactation: Official J Int Lactation Consultant Association.

[12] James Cole T (2002). Assessment of growth. Best Pract Res Clin Endocrinol Metab.

[13] Ashworth A, Shrimpton R, Jamil K (2008). Growth monitoring and promotion: review of evidence of impact. Matern Child Health.

[14] Feleke FW, Adole AA, Bezabih AM (2017). Utilization of growth monitoring and promotion services and associated factors among under two years of age children in Southern Ethiopia. PLoS ONE.

[15] Gyampoh S, Otoo GE, Aryeetey RNO (2014). Child feeding knowledge and practices among women participating in growth monitoring and promotion in Accra. Ghana.

[16] Sulley I, Abizari A-R, Ali Z, Peprah W, Yakubu HG, Forfoe WW (2019). Growth monitoring and promotion practices among health workers may be suboptimal despite high knowledge scores. BMC Health Serv Res.

[17] Appoh LY, Krekling S (2005). Maternal nutritional knowledge and child nutritional status in the Volta Region of Ghana. Maternal and Child Nutrition.

[18] Ghana Districts. A repository of all Local Assemblies in Ghana. (n.d.). Retrieved October 18., 2017, from http://ghanadistricts.gov.gh/DistrictSublinksaspx?s=1814&distID=139.

[19] WHO, UNICEF. Global strategy for infant and young child feeding. World Health Organization Publication; 2003.

[20] World Health Organization. Indicators for assessing infant and young child feeding practices: definitions and measurement methods [Internet]. Geneva, Switzerland: WHO. ; 2021 [Retrieved 2021 Jan 6]. Available from: https://apps.who.int/iris/bitstream/handle/10665/340706/ 9789240018389- eng.pdf?sequence = 1.

[21] Hall DM (2000). Growth monitoring. Arch Dis Child.

[22] Shrimpton R, Victora CG, de Onis M, Lima RC, Blössner M, Clugston G (2001). Worldwide timing of growth faltering: implications for nutritional interventions. Pediatrics.

[23] Charlton KE, Kawana BM, Hendricks MK. (2009). An assessment of the effectiveness of growth monitoring and promotion practices in the Lusaka district of Zambia. *Nutrition* 2009; *25*(10), 1035–1046. 10.1016/j.nut.2009.03.008. 10.1016/j.nut.2009.03.008 19660911

[24] Ijarotimi OS (2013). Determinants of Childhood Malnutrition and Consequences in developing countries. Curr Nutr Rep.

[25] Black RE, Allen LH, Bhutta ZA, Caulfield LE, de Onis M, Ezzati M, Maternal, and Child Undernutrition Study Group. (2008). Maternalchild undernutrition: globalregional exposureshealth consequences. *Lancet (London, England)* 2008; *371*(9608), 243–60. 10.1016/S0140-6736(07)61690-0. 10.1016/S0140-6736(07)61690-0 18207566

[26] Faber M, Phungula MA, Kvalsvig JD, Benadé AJ (2003). Acceptability of community-based growth monitoring in a rural village in South Africa. FoodNutr Bull.

[27] Yonas F (2015). Infant and young child feeding practice status and associated factors among mothers of under 24-month-old children in Shashemene Woreda, Oromia region, Ethiopia. Open Access Library Journal.

[28] WHO. Implementing the global strategy for infant and young child feeding: Geneva, 3–5 February 2003: meeting report. World Health Organization; 2003.

[29] Kabir I, Khanam M, Agho KE, Mihrshahi S, Dibley MJ, Roy SK (2012). Determinants of inappropriate complementary feeding practices in infant and young children in Bangladesh: secondary data analysis of Demographic Health Survey 2007. Matern Child Nutr.

[30] Senarath U, Agho KE, Akram DeS, Godakandage SS, Hazir T, Jayawickrama H (2012). Comparisons of complementary feeding indicators and associated factors in children aged 6–23 months across five south asian countries. Matern Child Nutr.

