# Supplementary material for: Utilization of growth monitoring and promotion services and undernutrition of children less than two years of age in Northern Ghana
Source: BMC Nutr. 2023 Jun 22;9:70. doi: 10.1186/s40795-023-00729-6 (PMC10286350; doi:10.1186/s40795-023-00729-6)
Supplement: Supplementary file 1 — Supplementary Material 1 [file 40795_2023_729_MOESM1_ESM.docx]

**QUESTIONNAIRE**

**GROWTH MONITORING AND PROMOTION, ITS ASSOCIATION WITH OPTIMUM NUTRITION CARE FOR CHILDREN AGED 0-23 MONTHS IN THE TAMALE METROPOLIS, GHANA**

**INFORMED CONSENT**

**IDENTIFICATION**

Date of interview…………………………………………

Sub-district name……………………………………………

Name of Community……………………………………

Name of Household Head……………………………

Questionnaire ID………………………………………………

Questionnaire Number…………………………………

**SECTION A; BACKGROUND CHARACTERISTICS**

| 1.How old are you? | 2. What is your relationship with child? |
| --- | --- |
| Response………………………… | 1.Mother  2.Father  3.Grandmother  4.Aunt  Other (specify)…………………………………. |

| **3.**How old is your child? | **4.**What is the birth weight of your child? |
| --- | --- |
| Response……………….***.(confirm from health card)*** | Response***…………………………(confirm from health card)*** |

| **5.**Sex of child. | **6.** How will you describe your current marital status? |
| --- | --- |
| 1.Male  2.Female | 1.Single  2.Married  3.Divorced  4.Seperated  5.Widowed |

| 7. Have you given birth before? | | 8. How many children do you have |
| --- | --- | --- |
| Yes  No | | One  Two  Three  Four  Five  Other (s), specify…………. |
| **9.**What is your highest level of education? | **10.**What is your main occupation? | |
| 1.None  2.Primary  3.Secondary  4.Tertiary | 1.Unemployed  2.Trader  3.Artisan  4.Buisnesswoman  5.Professional  Other(specify)……………………………… | |

| **11.** Where do you usually get advice from on how and what to feed your child? | **12.** What ethnic group do you belong? |
| --- | --- |
| 1.Family and friends  2.Media (TV/radio/print)  3.Internet  4.Health worker  Other (specify)………………………… | 1.Dagomba  2.Gonja  3.Mamprusi  4.Akan  Other (specify) …………………………….. |

| **13.** What is your type of tenancy? | **14.**What is your main source of energy for cooking? |
| --- | --- |
| 1.Caretaker  2.Company/government house  3.Rented  4.family house  5.Own house | 1.Firewood  2.charcoal  3.kerosene  4.LPG  5.Electric cooker |

**SECTION B; CHILD MORBIDITY**

| 15. Where do members of your household draw water from? | 16. Do you have any of the following in your household? |
| --- | --- |
| Borehole  Well  Surface water (e.g. river, stream, pond)  Piped water  Other(s), specify………… | Radio  Color/black and white TV  Satellite TV  Sewing machine  Mattress  Refrigerator  DVD/VCD/ Computer  Mobile phone  Motorcycle/tricycle  Car/truck |

| **17.**Has your child any special condition? | **18.**If yes, what type of special condition is it? |
| --- | --- |
| 1.Yes  2.No | 1.congenental (born with)  2.Not born with |

| **19.**Has the child had any sickness/illness in the past two weeks? | **20.**What do you do to your child when he/she fall sick? |
| --- | --- |
| 1.Yes  2.No | 1.Take the child to the hospital  2.Purchase drugs at the pharmacy/chemical shop  3.Private medical assistant  4.Nothing  Other (specify)………………………………. |

**SECTION C; FEEDING PRACTICES**

***Caregivers with children 0-5 completed months***

***(Please fill in the boxes provided to indicate answers where applicable)***

21.Was yesterday a typical day for the child? If yes, please describe the foods (meals and snacks) that the child ate or drank yesterday during the day and night, whether at home or outside the home. Start with the first food or drink of the morning. Write down all foods and drinks mentioned including breast milk. When composite dishes are mentioned, ask for the list of ingredients. When the respondent has finished, probe for meals and snacks not mentioned.

| **Breakfast** | **Snack** | **Lunch** | **Snack** | **Dinner** | **Snack** |
| --- | --- | --- | --- | --- | --- |
|  |  |  |  |  |  |

| **22.** If child was given anything other than breast milk, why? | **23.**How many times do you breastfeed your child within 24hrs.? |
| --- | --- |
| 1.Started work  2.breastmilk insufficient  3.Child cannot suckle  4.Painful breastfeeding  5.Maternal illness  6.New pregnancy  Other (specify)………………………. | 1.6-10 times  2.8-12 times  3.5-9 times  4.>12 times |

| **24.**What do you observe of your child to suggest that he/she needs to be fed? | **25.**At what time of the day do you breastfeed more? |
| --- | --- |
| 1.crying  2.restlessness  3.sucking of thump  4.opening mouth to bring out tongue  Other (specify)………………………….. | 1.Day time  2.At night  Other (specify)……………………………. |

| **26.**How often do you switch breast for your child during feeding time? | **27.**How do you position your baby to breastfeed? ***(observe and tick below).*** |
| --- | --- |
| 1.Every 30 mins  2.When weight of breast reduces  3.when I want to work on either side of my breast  Other (specify)……………………………… | 1.Baby in line  2.Baby close to mother  3.Babys whole body supported  4.Baaby facing mother |

| **28.**At what age (months) did the child receive water for the first time? |  |
| --- | --- |
| 1.<3 months  2.4-5 months  3.Not yet |  |

***Caregivers with children 6-23months***

***(Please tick or fill in the boxes provided to indicate answers where applicable)***

29.Was yesterday a typical day for the child? If yes, please describe the foods (meals and snacks) that the child ate or drank yesterday during the day and night, whether at home or outside the home. Start with the first food or drink of the morning. Write down all foods and drinks mentioned including breast milk. When composite dishes are mentioned, ask for the list of ingredients. When the respondent has finished, probe for meals and snacks not mentioned.

| **Breakfast** | **Snack** | **Lunch** | **Snack** | **Dinner** | **Snack** |
| --- | --- | --- | --- | --- | --- |
|  |  |  |  |  |  |

| **30.** If child did not receive other foods at all in addition to breast milk, why? | **31.**At what age did child receive water for the first time? |
| --- | --- |
| 1.It is not yet time to introduce other foods  2.child refuses to eat  3.child was sick  4.I don’t know what to give  5.Work constraint  Other (specify)………………………… | 1.<3 months  2.4-5 months  3.At 6 months  4.After 6 months |

| **32.** At what age did the child receive other foods for the first time? |  |
| --- | --- |
| 1.<3 months  2.4-5 months  3.At 6 months  4.After 6 months |  |

**SECTION D; CHILD FEEDING KNOWLEDGE**

| **33.** For how long can a child be breast fed after introducing other foods? |  |
| --- | --- |
| 1.0-5 months  2.6-11 months  3.12-17 months  4.18-23 months  5.>24 months  6.dont know/not sure |  |

**Measured knowledge item**

| **Food group** | **Age of introduction** | **For each food group**  **Introduction at 6-8 months =1.**  **Introduction before or after this age range = 0** |
| --- | --- | --- |
| **34.** Water/other liquids |  | [ ] |
| **35.** Staple foods (cereals, roots and tubers) |  | [ ] |
| **36.** Vegetables (added to food or on their own) |  | [ ] |
| **37.** Fruits |  | [ ] |
| **38.** Dairy products (milk, cheese, yoghurt etc) |  | [ ] |
| **39.** Eggs (yolk and whole egg) |  | [ ] |
| **40.** Meats (chicken , fish, meat) |  | [ ] |
| ***Go to questions 30-32 if child is 6-23 months old*** |  |  |
| **Appropriate feeding frequency (please fill in per the age of caregiver’s child)** |  |  |
| **41.** No. of meals/day for 6-8 month old breastfeeding child |  | 0-1 meal/day=0, 2 and higher=1 [ ] |
| **42.** No. of meals/day for 9-23 month old breastfeeding child |  | 0-2 meals/day=0, 3 and higher=1 [ ] |
| **43.** No. of meals/day for 6-23 month old non-breastfed child |  | 0-3 meals/day=0, 4 and higher=1 [ ] |

**SECTION E; KNOWLEDGE AND ATTITUDE TOWARDS GMP**

***(GMP: Growth Monitoring and Promotion)***

| **44.** What is the number of months you attended Child Welfare Clinic from birth of child? ***(refer to health card***) | **45.**What is the number of months missed from birth of child? (***refer to health card***) |
| --- | --- |
| RESPONSE……………………... | RESPONSE…………………………………. |

| **46.**If any months were missed, what were the barriers to your attendance? |
| --- |
| 1.Transport cost  2.Travelled  3.Work  Other (specify)……………………………. |

| **47.**Do consider monthly weighing of your child important? | **48.**If yes, what importance is it to you? |
| --- | --- |
| 1.Yes  2.NO | 1.To monitor child’s weight/growth  2.To know if child is healthy  3.To seek medical care where necessary  4.To seek nutritional advice where necessary  Other (specify)………………………… |

***Use the sample growth charts provided as illustrations to assess the caregiver’s comprehension from the questions below.***

| **49.** What is the purpose of the growth chart? | **50.** What does it mean for a child when the curve on the growth chart is falling? |
| --- | --- |
| Monitoring/recording growth/weight of children  don’t know | Child is not growing well/has lost weight  child maybe sick/has been sick  Child is not eating well  don’t know  other (specify)………... |

| **51.** What will you do for the child in the case above (question 18) | **52.** What does it mean for a child when the curve on the growth chart is flattening? |
| --- | --- |
| Feed the child better/more  take child to hospital  seek nutritional care  Don’t know  Other (specify) ……………………. | Child is not growing well/has not gained enough weight  Child maybe sick/has been sick  Child is not eating well  Don’t know  0ther (specify)……………………… |

| **53.** What will you do for the child in the case above (question 20) | **54.** What does it mean for a child when the curve on the growth chart is rising? |
| --- | --- |

| **55.** What will you do for the child in the case above (question 22) | **56.** Do you intend to continue attending CWC after the child is fully immunized? |
| --- | --- |
| Continue to feed and care for child well  don’t know  Other (specify)…………………. | 1.YES  2.NO |

**SECTION E; CHILD ANTHROPOMETRIC DATA**

| **57.** Weight: | **58.** Length/Height: |
| --- | --- |
| 1^st^ measurement ( )kg  2^nd^ measurement ( )kg  Average ( ) kg | 1^st^ measurement ( )cm  2^nd^ measurement ( )cm  Average ( )cm |

**END OF INTERVIEW, THANK RESPONDENT**
